# Demonstrating the Theory of Ecological Speciation in Cichlids

**DOI:** 10.1371/journal.pbio.0040449

**Published:** 2006-12-05

**Authors:** Liza Gross

The geological history of Africa’s Lake Victoria, the second largest freshwater lake in the world, provided the raw materials for investigating one of the most compelling hypotheses for the origin of species: ecological speciation. After drying out three times over its 400,000-year history, the lake refilled about 15,000 years ago, and the few cichlid fish species that had retreated to fluvial habitats returned, rapidly fanning out into hundreds of new species to fill different ecological niches.

Though Lake Victoria cichlids appear millions of years younger than their counterparts in nearby Lake Malawi, both groups display an enormous range of physical and behavioral traits. This staggering diversity in such young species provides compelling evidence for adaptive radiation, which occurs when divergent selection operates on ecological traits that favor different gene variants, or alleles, in different environments. When divergent selection on an ecological trait also affects mate choice—promoting reproductive isolation of diverging populations—ecological diversity and speciation may proceed in tandem and quickly generate numerous new species.

Despite substantial theoretical and some experimental support for such “by-product speciation,” few studies have shown that selection has “fixed” alleles (that is, driven its frequency in a population to 100%) with different effects on an adaptive trait in closely related populations. But now, Yohey Terai, Norihiro Okada, and their colleagues have bridged that gap by demonstrating divergent selection on a visual system gene that influences both ecological adaptation and mate choice in cichlids.

Photoreceptors in the retina perceive light with visual pigments that consist of a light-absorbing chromophore (either A1 or A2) that sits inside an opsin protein. The chromophore interacts with several amino acids coating the opsin to determine the pigment’s light sensitivity. In cichlids, opsins with the most variable sequences function at the opposite ends of the light spectrum: the short wavelength–sensitive opsin 1 *(SWS 1)* perceives ultraviolet blue, and the long wavelength–sensitive opsin *(LWS)* perceives red. Because *LWS* shows five times more variation in Lake Victoria cichlids than it does in Lake Malawi cichlids—and species’ spectral range matches male breeding coloration, a primary determinant in mate choice—the authors suspected the gene might simultaneously affect ecological adaptation and mate choice.

**Figure:**
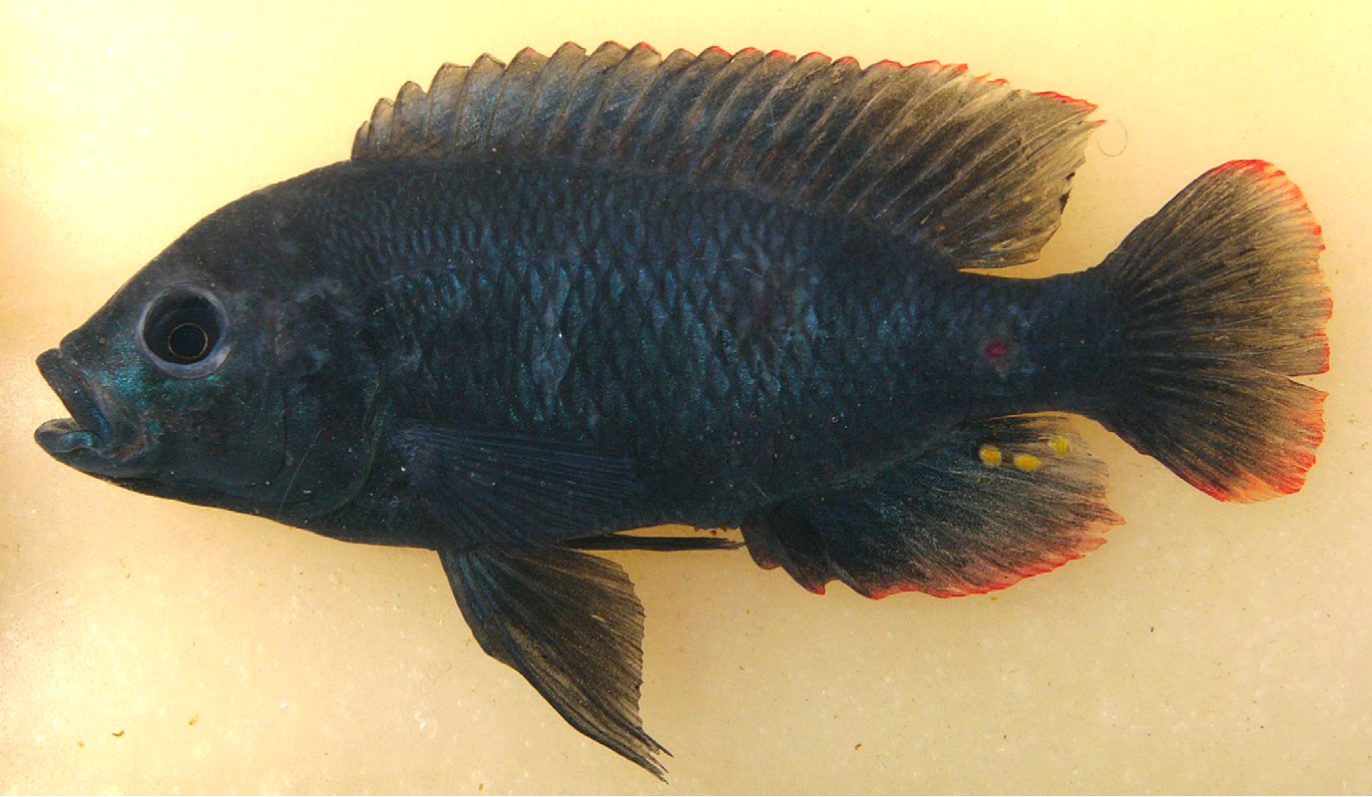
The rapid evolution of African cichlid fish driven by strong divergent selection is revealed in a gene that influences both ecological adaptation and mate choice, in keeping with ecological by-product speciation.

The authors sequenced hundreds of *LWS* alleles from four Lake Victoria cichlid species inhabiting different microhabitats. Both *Mbipia mbipi* and *Neochromis greenwoodi/N. omnicaeruleus* (grouped together based on their similar characteristics) live outside rocky crevices in the lake’s turbid depths, though their depth ranges differ. *N. rufocaudalis* also lives outside rocky crevices, but inhabits shallow waters like *Pundamilia pundamilia*, which live among the crevices. Transparent waters transmit broad spectra; turbid waters shift the visual spectrum toward red. The authors predicted that populations of the deeper-living species—*N. greenwoodi/N. omnicaeruleus* and *M. mbipi*—would be affected by light transmission with different water clarity (which was not an issue for those living in shallow waters).

They focused on *LWS* polymorphisms in opsin amino acids that would alter light sensitivity, grouping them into L and H alleles. L alleles were fixed (or nearly so) in turbid-water dwelling populations; H alleles were fixed in populations accustomed to transparent water. Finding a strong positive correlation between *LWS* divergence and transparency, the authors determined that significant differentiation in *LWS* sequences (that is, population variation in allele frequencies) resulted from divergent selection. And, as expected, they found only weak sequence differentiation between the populations in shallow, transparent waters. Divergent selection acted on the *LWS* alleles only between *N. greenwoodi/N. omnicaeruleus* and *M. mbipi* populations from different water transparencies, the authors concluded, “strongly implicating divergent adaptation to different photic environments.”

To test the adaptive implications of divergence, the authors reconstituted pigments from H and L alleles along with A1 or A2 chromophores and measured their light-absorption range. A1 pigments absorbed the same spectra in H and L alleles, but the A2 pigment caused a red shift only in the L allele—likely reflecting an adaptation to the longer wavelengths found in turbid waters. And how did divergent light sensitivity compare with male breeding color? The populations that diverged according to water transparency also diverged in male breeding coloration—some *N. greenwoodi/N. omnicaeruleus* males are yellow-red and some *M. mbipi* are yellow. In turbid waters, both yellow and red travel farther than blue light, and the populations with alleles shifted toward the longer yellow and red wavelengths had a higher frequency of males with corresponding yellow-red or yellow males. Why all males haven’t evolved red and yellow breeding coloration is a question the authors are currently studying.

Altogether, these results demonstrate that by-product speciation—driven by strong divergent selection in a gene controlling an ecological trait that affects mate choice—fueled the rapid evolution of African cichlids. The colorful cichlids have proven invaluable in illuminating the mechanisms of speciation. But biologists now face a race against time to plumb their secrets: an estimated 50% of cichlids vanished in the 1980s and appear to be disappearing ten times faster than they can be described.

